# Are There any Relationships between Latent *Toxoplasma gondii* Infection, Testosterone Elevation, and Risk of Autism Spectrum Disorder?

**DOI:** 10.3389/fnbeh.2014.00339

**Published:** 2014-09-24

**Authors:** Amir Abdoli, Abdolhossein Dalimi

**Affiliations:** ^1^Faculty of Medical Sciences, Department of Parasitology, Kashan University of Medical Science, Kashan, Iran; ^2^Faculty of Medical Sciences, Department of Parasitology, Tarbiat Modares University, Tehran, Iran

**Keywords:** *Toxoplasma gondii*, prenatal testosterone, autism, extreme male brain theory, latent infection, second to fourth digit ratio, sex ratio

## Autism Spectrum Disorder and the “Extreme Male Brain” Theory

Autism spectrum disorder (ASD) is a set of complex neurodevelopmental disorders. ASD is characterized by early-onset difficulties in social interaction, repetitive behavior, and verbal and non-verbal communication. Worldwide prevalence of ASD is about 1% (Lai et al., 2014). Several factors have been proposed in the etiology of ASD, including genetics background, obstetric complications, intrauterine infections, environmental conditions, immune imbalance, and fetal testosterone levels (Rutter, 2005; Currenti, 2010; Ratajczak, 2011; Gesundheit et al., 2013; Goines and Ashwood, 2013; Jyonouchi, 2013; Lai et al., 2014). Autism affects males more frequently than females (Baron-Cohen et al., 2011; Werling and Geschwind, 2013; Developmental Disabilities Monitoring Network Surveillance Year 2010 Principal Investigators and Centers for Disease Control and Prevention, 2014), with ratios of 4:1 (male: female) for classic autism (Chakrabarti and Fombonne, 2001) and 11:1 in individuals with Asperger syndrome (AS) (Gillberg et al., 2006). This evidence raises an important question: what influencing factors are responsible for the higher male prevalence of autism? The exact answer to this question remains unclear; however, the “extreme male brain” theory of autism (Baron-Cohen, 2002) proposed that exposure to high levels of prenatal testosterone is one possible mechanisms (Baron-Cohen, 2002; Baron-Cohen et al., 2011).

## Latent Toxoplasmosis Influence Testosterone Production

Toxoplasmosis is one of the most common zoonotic diseases that has infected approximately one-third of the world’s human population (Montoya and Liesenfeld, 2004). Although it is estimated between 25 and 30% of the world’s human population is infected by *T. gondii*, and the most common form of infection is latent (asymptomatic) (Dalimi and Abdoli, 2012; Robert-Gangneux and Dardé, 2012). On the other hand, latent toxoplasmosis can induce different hormonal and behavioral alterations in humans and rodents (Flegr, 2013a,b) and involved in the etiology of various psychotic disorders (Dalimi and Abdoli, 2012; Flegr, 2013a; Abdoli et al., 2014). Different studies reported an increased concentration of testosterone in men with latent toxoplasmosis compared to the testosterone in *Toxoplasma*-negative individuals (Flegr et al., 2008a,b; Shirbazou et al., 2011; Zghair et al., 2014). Increased concentrations of testosterone were also confirmed in an animal model of latent toxoplasmosis (Lim et al., 2013). In addition, several indirect evidences exist for increased prenatal testosterone in latent toxoplasmosis (see the next sections). This condition raises an important question: does latent toxoplasmosis increase risk of ASD via mechanisms consistent with the “extreme male brain” theory of autism? If this hypothesis is correct, pregnant women with latent toxoplasmosis are more likely to give birth to a child with ASD. Hence, we summarize the evidences of increased prenatal testosterone in ASD and latent toxoplasmosis to offer a new insight into the role of *T. gondii* infection in the etiology of ASD.

## Direct Evidences for Increased Prenatal Testosterone in Autistic-Like Traits and ASD

There is both direct and indirect evidence for the role of prenatal testosterone and related androgens in the etiology of autistic traits and ASD. Recently, Baron-Cohen et al. (2014) found direct evidence of elevated fetal steroidogenic activity in ASD individuals from the Danish Historic Birth Cohort. In this study, concentrations of Δ4 sex steroids (testosterone, androstenedione, 17α-hydroxy-progesterone, and progesterone) and cortisol were measured in amniotic fluid samples of 128 males born between 1993 and 1999 who later received ICD-10 (International Classification of Diseases, 10th Revision) diagnoses of autism, AS or PDD-NOS (pervasive developmental disorder not otherwise specified), compared with healthy controls. The results showed significant elevations of all hormones in ASD individuals (Cohen’s *d* = 0.37, *p* = 0.0009).

Autistic-like traits are the first signs of ASD (Lundström et al., 2012). Moreover, individuals with one or more autistic traits also appear to be at higher risk for ASD, attention deficit hyperactivity disorder (ADHD), depression, and anxiety (Lundström et al., 2011). In this regard, a positive correlation between prenatal testosterone levels and higher scores on the childhood autism spectrum test (CAST) and Child Autism Spectrum Quotient (AQ-Child) were reported from the Cambridge Fetal Testosterone Project, which measured prenatal testosterone levels during amniocentesis and compared the numbers of autistic traits in toddlers (Auyeung et al., 2009, 2010). Moreover, boys scored higher on the Quantitative Checklist for Autism in Toddlers (Q-CHAT) than girls (Auyeung et al., 2009). Auyeung et al. (2012) compared the effects of prenatal versus postnatal testosterone and estradiol levels on the number of autistic traits among children between 18 and 24 months of age. The results of the Q-CHAT were positively associated with the levels of prenatal testosterone (*p* < 0.05) (but not postnatal testosterone or either prenatal or postnatal estradiol levels) (Auyeung et al., 2012). Another study by Xu and colleagues (Xu et al., 2013) revealed a significant higher level of plasma testosterone (*p* < 0.05) in mothers of autistic children compared to control subjects.

Congenital adrenal hyperplasia (CAH) is a disease that leading to overproduction of adrenal androgens due to an enzyme deficiency [usually of 21 hydroxylase (21-OH)] (Knickmeyer et al., 2006). Knickmeyer et al. (2006) reported that the total scores of AQ were significantly higher in females with CAH compared with unaffected females.

## Indirect Evidences for Increased Prenatal Testosterone in ASD and Latent Toxoplasmosis

### Second to fourth digit ratio

It is well documented that the ratio between the length of the second digit (the “index” finger) and the fourth digit (the “ring” finger) is an indicator of prenatal sex steroid levels (Manning, 2011; Zheng and Cohn, 2011; Manning et al., 2014). However, the role of adult sex steroids in this ratio is less clear (Hönekopp et al., 2007). The ratio of 2D:4D is a sexually dimorphic trait that is different in male and female; it means that the ring finger relative to index finger is longer among males (low 2D:4D) than females (high 2D:4D) (Hönekopp and Watson, 2010). 2D:4D ratio in males is negatively related to testosterone level; and in both sexes is positively related to estrogen level (Manning et al., 1998; Manning and Bundred, 2000; Lutchmaya et al., 2004). It has been observed that 2D:4D ratio is related to several neuropsychiatric disorders, including; ASD (De et al., 2006), schizophrenia (Arató et al., 2004; Collinson et al., 2010), anxiety, and depressive disorders (Bailey and Hurd, 2005a), as well as different behavioral parameters (Neave et al., 2003; Bailey and Hurd, 2005b) and morphometric traits (Fink et al., 2004, 2005; Burriss et al., 2007).

#### 2D:4D ratio in individuals with ASD

Several studies have investigated 2D:4D ratios among individuals with ASD, and the majorities of them reported lower 2D:4D ratios in autistic probands [reviewed in Teatero and Netley (2013)]. For example, Manning et al. (2001) reported that 2D:4D ratios of autistic children, their siblings, and parents were lower than the ratio of the normal population. The results of a recent meta-analysis revealed that digit ratios of individuals with ASD are lower (0.10–0.77 standard deviations) than control subjects (Teatero and Netley, 2013).

Attention deficit hyperactivity disorder is a group of behavioral symptoms including hyperactivity, inattention, and impulsiveness. ADHD symptoms are often observed in individuals with ASD. Both disorders are more frequent in males than females (Ames and White, 2011; Davies, 2014), and it is plausible that both disorders may be related to high prenatal testosterone levels (James, 2008a). Recently, Romero-Martínez et al. (2014) investigated the relationships between ASD and ADHD symptoms through measurement of 2D:4D ratio, salivary testosterone levels, and inattention behavior of 32 parents with ASD children and their offspring. They observed that inattentive ADHD symptoms in ASD parents and their offspring were significantly associated with masculinized 2D:4D and increased salivary testosterone levels (Romero-Martínez et al., 2014).

#### 2D:4D ratio in individuals with latent toxoplasmosis

In addition to the direct effects of latent toxoplasmosis on testosterone concentration, there are also some phenotypic traits in individuals with latent toxoplasmosis that related to higher levels of prenatal testosterone (Flegr et al., 2008b; Flegr, 2010, 2013b). For example, both men and women with latent toxoplasmosis have a lower 2D:4D ratio than non-infected subjects (Flegr et al., 2005). *T. gondii*-infected men are taller in stature than non-infected men (Flegr et al., 2005). The faces of infected men in photographs are more masculine and dominant than non-infected subjects when rated by women raters (Hodková et al., 2007). Unlike these findings, Manning and Fink (2011) found no significant correlations between mean national scores of 2D:4D and prevalence of *T. gondii* among 23 nations (Manning and Fink, 2011). On the other hand, Lafferty (2006) found positive relationships between individual personality scores and *T. gondii* prevalence among different countries. These contradictory results seem to be influenced by different factors; including infection with different strains of *T. gondii*, host variations in susceptibility to infection, duration and intensity of *T. gondii* infection. Consequently, these influencing factors have diverse effects on testosterone production, behavioral alterations, and neuropsychiatric disorders (Abdoli, 2013, 2014).

### Sex ratio in individuals with ASD and latent toxoplasmosis

The ratio of boys to girls at birth is around 0.51 in most populations. Several factors, including maternal endocrine disruption, stress, and immunosuppression are believed influence this ratio (James, 2008b). According to the hormonal hypothesis (James, 2008b, 2014), high levels of testosterone in both parents and estrogen levels in mothers, around the time of conception, are associated with a higher production of sons; high levels of gonadotrophins in both parents are associated with a higher production of daughters (James, 2008b, 2014).

#### Sex ratio in individuals with ASD

It is very well documented that the prevalence of ASD in males are more frequent than females (Chakrabarti and Fombonne, 2001; Gillberg et al., 2006; Baron-Cohen et al., 2011; Werling and Geschwind, 2013; Developmental Disabilities Monitoring Network Surveillance Year 2010 Principal Investigators and Centers for Disease Control and Prevention, 2014), with ratios of 4:1 (male: female) for ASD (Chakrabarti and Fombonne, 2001) and 11:1 in AS (Gillberg et al., 2006). The result of recent surveillance in the United States reveals that prevalence of ASD is approximately one in 42 boys and one in 189 girls (Developmental Disabilities Monitoring Network Surveillance Year 2010 Principal Investigators and Centers for Disease Control and Prevention, 2014).

#### Sex ratio in individuals with latent toxoplasmosis

It has been observed that the sex ratio was significantly increased in pregnant women with high levels of anti-*Toxoplasma* IgG antibodies (proportion of males: 0.608 in *Toxoplasma* positive and 0.527 in *Toxoplasma*-negative mothers; *p* = 0.0027); in contrast, the sex ratio was significantly decreased in pregnant women with low levels of anti-*Toxoplasma* IgG antibodies (Kaňková et al., 2007a). These observations have been confirmed by the same group of researchers in a mouse model of toxoplasmosis, in which the sex ratio was increased at the early phases of infection, but then decreased in later phases (Kaňková et al., 2007b).

### Testosterone-related medical disorders in individuals with ASD and latent toxoplasmosis

Ingudomnukul et al. (2007) reported that women suffering from ASD had significantly higher rates of testosterone-related medical disorders (including hirsutism, bisexuality or asexuality, irregular menstrual cycle, dysmenorrhea, polycystic ovary syndrome, severe acne, epilepsy, tomboyism, and family history of ovarian and uterine cancers) compared to controls. Interestingly, mothers of children with ASD had significantly more severe acne, breast and uterine cancers, and family history of ovarian and uterine cancers, compared to controls (Ingudomnukul et al., 2007). In latent toxoplasmosis, Shirbazou et al. (2011) reported a significantly higher concentration of testosterone in women with latent toxoplasmosis than in *Toxoplasma*-negative women (*p* = 0.002). Moreover, infected women had significantly more facial hair (*p* = 0.001) and hair reduction (*p* = 0.002) than the control group.

Several complex neurodevelopmental disorders may be associated with higher levels of testosterone, including antisocial and aggressive behavior (Rowe et al., 2004; Ingudomnukul et al., 2007; Eisenegger et al., 2011; Romero-Martínez et al., 2014), bipolar disorder, suicidal behavior (Sher et al., 2012, 2014; Sigurdsson et al., 2014), and ADHD symptoms (Romero-Martínez et al., 2014). Interestingly, some of these disorders have been reported in individuals with latent toxoplasmosis and ASD probands. For example, ASD probands have sometime represented aggressive behaviors, self-injurious behaviors, and suicidal attempts (Lai et al., 2014). On the other hand, several testosterone-related behavioral abnormalities (Flegr, 2007), as well as self-injurious and suicidal attempts (Arling et al., 2009; Ling et al., 2011; Pedersen et al., 2012; Zhang et al., 2012) have been reported in individuals with latent toxoplasmosis.

## Other Possible Mechanisms of *T. gondii* That Could Contribute in the Etiology of ASD

Different mechanisms have been shown to involve in the etiology of neuropsychiatric disorders and behavioral alterations during *T. gondii* infection, including hormonal alterations [increased testosterone (Flegr et al., 2008a,b; Lim et al., 2013)], neurotransmitters manipulation [particularly increase dopamine and decrease serotonin (Stibbs, 1985; Skallova et al., 2006; Gaskell et al., 2009; Prandovszky et al., 2011)], decrease tryptophan and increase kynurenic acid (Schwarcz and Hunter, 2007; Notarangelo et al., 2014), and various immunological alterations (Prandota, 2010a,b, 2011). *T. gondii* infection also induces different abnormalities in specific regions of the brain (e.g., hippocampus and amygdala) that involved in the etiology of various neuropsychiatric disorders (Vyas et al., 2007; Hermes et al., 2008; Gatkowska et al., 2012; Mitra et al., 2013; Evans et al., 2014). Interestingly, increased dopamine levels were reported in individuals with ASD (Gillberg and Svennerholm, 1987; Previc, 2007; Nakamura et al., 2010) and AS (Nieminen-von Wendt et al., 2004). On the other hand, testosterone is also linked to dopamine (Hull et al., 1995, 2004) and both are increased during latent toxoplasmosis (Stibbs, 1985; Skallova et al., 2006; Gaskell et al., 2009; Prandovszky et al., 2011; Xiao et al., 2013). It is also observed that the connection between testosterone and dopamine is mediated by nitric oxide (NO) (Hull et al., 2004); while, increased testosterone enhances dopamine levels (Hull et al., 1995). Moreover, NO and other inflammatory cytokines such as Interleukin-2 (IL-2) and IL-6 are innate defenses against *T. gondii* infection (Miller et al., 2009; Yarovinsky, 2014). NO, IL-2, and IL-6 also increase dopamine release (Alonso et al., 1993; Zalcman et al., 1994; Petitto et al., 1997; Prast and Philippu, 2001; West et al., 2002).

## Conclusion and Future Directions

Several direct and indirect evidences support the role of increased prenatal testosterone in ASD and latent toxoplasmosis. Latent toxoplasmosis may also be involved in the etiology of various testosterone-related medical disorders and plays different roles in the etiology of neuropsychiatric disorders. Taken together with the high prevalence of latent toxoplasmosis in different nations (approximately 25–30% of the world’s human population (Robert-Gangneux and Dardé, 2012); therefore, increased prevalence of ASD as well as different testosterone-related medical disorders are postulated in nations with higher prevalence of toxoplasmosis. However, more research is needed to clarify the role of latent toxoplasmosis in the etiology and epidemiology of ASD and related disorders.

## Conflict of Interest Statement

The authors declare that the research was conducted in the absence of any commercial or financial relationships that could be construed as a potential conflict of interest.
